# *Wolbachia* introgression in Rio de Janeiro remains at sub-optimal levels 30 months after its decline: challenges in the sustainability of *w*Mel interventions for dengue control

**DOI:** 10.1590/0074-02760250212

**Published:** 2026-10-05

**Authors:** Karine Pedreira Padilha, Gabriela Azambuja Garcia, Rayane Teles-de-Freitas, Franck Jeannot Gnonhoue, Mariana Rocha David, Jessica Corrêa-Antônio, Felipe de Oliveira, Luiz Paulo Brito, Luciana Dias, Ademir Jesus Martins, Vincent Corbel, José Bento Pereira Lima, Gabriel Luz Wallau, Ary Hoffmann, Oswaldo Gonçalves Cruz, Daniel Antunes Maciel Villela, Márcio Galvão Pavan, Rafael Maciel-de-Freitas

**Affiliations:** 1 Fundação Oswaldo Cruz-Fiocruz, Instituto Oswaldo Cruz, Laboratório de Mosquitos Transmissores de Hematozoários, Rio de Janeiro, RJ, Brasil; 2 Fundação Oswaldo Cruz-Fiocruz, Instituto Oswaldo Cruz, Laboratório de Biologia de Tripanosomatídeos, Rio de Janeiro, RJ, Brasil; 3 Fundação Oswaldo Cruz-Fiocruz, Instituto Oswaldo Cruz, Laboratório de Biologia, Controle e Vigilância de Insetos Vetores, Rio de Janeiro, RJ, Brasil; 4 University of Montpellier, Centre National de la Recherche Scientifique, Institut de Recherche pour le Développement, Maladies Infectieuses et Vecteurs Écologie Génétique Évolution et Contrôle, Montpellier, France; 5 Fundação Oswaldo Cruz-Fiocruz, Instituto Aggeu Magalhães, Departamento de Entomologia e Bioinformática, Recife, PE, Brasil; 6 Bernhard Nocht Institute for Tropical Medicine, Department of Entomology and Arbovirology, Hamburg, Germany; 7 Universidade Federal de Santa Maria, Santa Maria, RS, Brasil; 8 The University of Melbourne, Bio21 Institute, School of BioSciences, Pest and Environmental Adaptation Research Group, Melbourne, Australia; 9 Fundação Oswaldo Cruz-Fiocruz, Programa de Computação Científica, Rio de Janeiro, RJ, Brasil

**Keywords:** Aedes aegypti, Wolbachia, *w*Mel, arboviruses control, dengue, insecticide resistance, integrative vector management

## Abstract

**BACKGROUND:**

The deployment of the *Wolbachia w*Mel strain is currently underway in multiple dengue-endemic municipalities across Brazil. The efficacy of this strategy in Rio de Janeiro remains uncertain, primarily due to the difficulty in sustaining high *w*Mel prevalence in regions previously subjected to large-scale releases. A key contributing factor was the routine rotation of insecticides within the framework of Integrated Vector Management (IVM), which led to the use of the larvicide Spinosad for *Aedes* control in urban areas. This compound was associated with a precipitous decline in *Aedes aegypti* mosquitoes, regardless of the *w*Mel infection status. While *w*Mel-uninfected population recovered within weeks, *w*Mel-infected population remained at low levels likely due to the fitness costs imposed by *w*Mel on egg viability.

**OBJECTIVES and METHODS:**

Evaluate the updated frequency of *Wolbachia w*Mel strain in neighborhoods that received deployments. Fort that, we assessed the long-term persistence of *w*Mel following its demographic collapse, by performing mosquito sampling across 12 neighbourhoods in Rio de Janeiro, 30 months after mosquito populations crashed.

**FINDINGS:**

Our findings reveal that *w*Mel introgression remains suboptimal, with a mean frequency of 9.87% across the sampled areas. Only two neighbourhoods exhibited *w*Mel frequencies exceeding 15%, likely reflecting ongoing localised releases.

**MAIN CONCLUSIONS:**

The reduced prevalence underscores the challenges of achieving self-sustaining *w*Mel establishment in complex urban environments and highlight critical considerations for the implementation of *Wolbachia*-based dengue control programs in endemic regions.

*Aedes aegypti* (Diptera: Culicidae) is a mosquito species extensively distributed across urban areas of tropical and subtropical regions. It constitutes a major public health concern as the principal vector of several medically important arboviruses, including dengue, Zika, and Chikungunya viruses. Among these, dengue virus (DENV) is particularly significant due to its widespread global presence and the frequent outbreaks and epidemics it causes, affecting millions of individuals annually.^1^ ^2^ The burden of dengue is predominantly concentrated in tropical regions, with Brazil consistently reporting the highest number of cases.^3^ In 2024 alone, Brazil registered over 6.6 million suspected dengue cases, marking the highest annual incidence since the virus was reintroduced into the country in the late 1980s - early 1990s.^4^

The continued rise in arboviral cases globally, and particularly in Brazil, demonstrates the limited effectiveness of conventional vector control methods, such as chemical insecticides and mechanical interventions.^5^ ^6^ ^7^ This scenario underscores the urgent need for complementary strategies to enhance *Ae. aegypti* population control.^8^ ^9^ Among several innovative approaches, the use of the symbiotic bacterium *Wolbachia pipientis* has gained attention. While naturally absent in *Ae. aegypti*, *Wolbachia* strains have been successfully transinfected from other insect species into *Ae. aegypti*, and some of them have pathogen-blocking properties.^10^ ^11^ ^12^ ^13^ ^14^ The spread of *Wolbachia* strains through arthropod populations is favoured by a combination of efficient maternal transmission, which is dependent on high *Wolbachia* density in female reproductive systems, and their ability to cause cytoplasmic incompatibility (CI), in which uninfected females are at a disadvantage over infected females because they fail to produce offspring when mated with infected males.^15^ It is thereby possible to replace local *Ae. aegypti* populations with high susceptibility to arbovirus with *Wolbachia*-infected equivalents that have a reduced potential for pathogen transmission.

The successful introgression of *Wolbachia* into native *Ae. aegypti* populations involves reaching a high prevalence in the target area with long-term stability. Monitoring efforts are essential to evaluate the proportion of mosquitoes carrying *Wolbachia*, thereby assessing the effectiveness and persistence of the introgression. Notably, *Wolbachia* prevalence in *Ae. aegypti* populations can fluctuate over time and often displays a markedly heterogeneous spatial distribution.^16^ ^17^ ^18^ ^19^ ^20^ These fluctuations may be driven by several factors, including reduced fitness of *Wolbachia*-infected individuals, insecticide resistance, migration of wild-type mosquitoes, and environmental conditions such as high temperatures, which can particularly affect the stability of the *w*Mel strain of *Wolbachia*.^21^ ^22^ ^23^ ^24^ ^25^ ^26^ ^27^ ^28^ ^29^ ^30^ ^31^

**Fig. 1:**
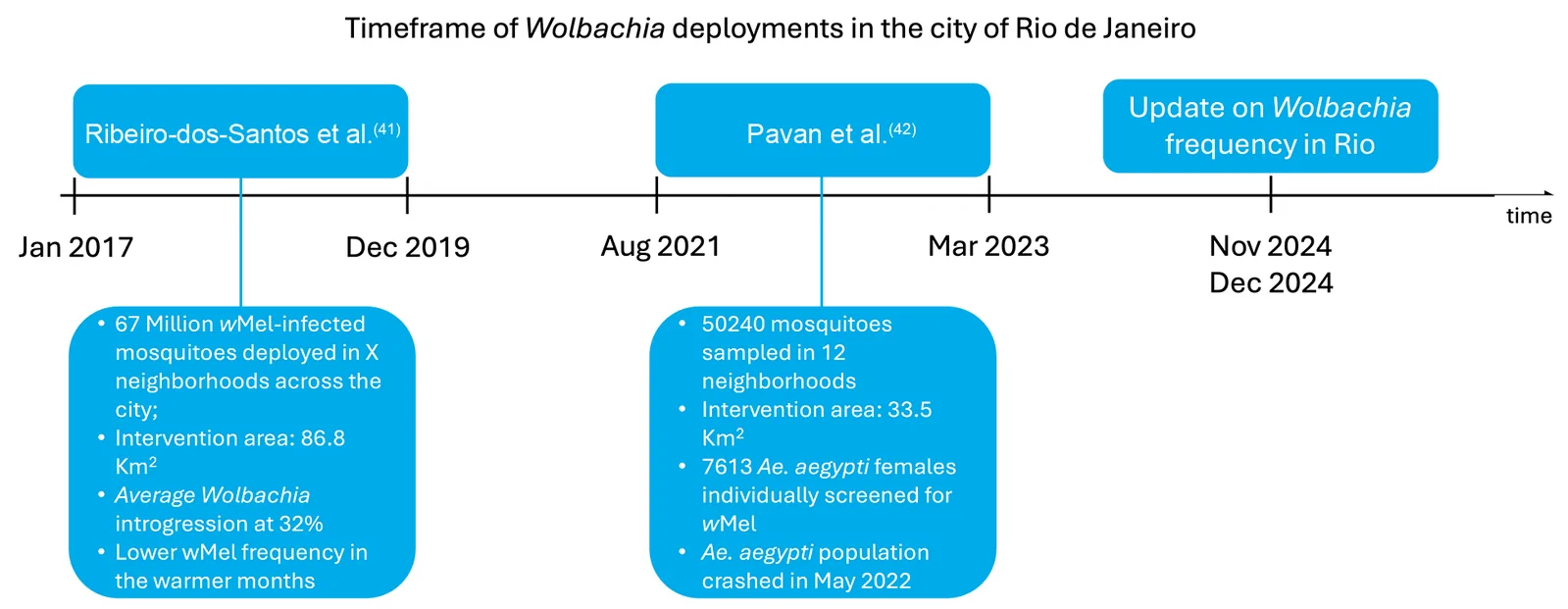
timeline of *w*Mel deployment and monitoring in the city of Rio de Janeiro, with critical information available in the two previous periods: Jan/2017 to Dec/2019^41^ and Aug/2021 to Mar/2023.^42^ The *Aedes aegypti* population crashed in May 2022, likely due to an increasing coverage of Spinosad larvicide in the study area.^42^

In the last ten years, *Wolbachia* deployments have been conducted in several cities worldwide from countries as diverse as Australia, Brazil, Colombia, Indonesia, Malaysia, Singapore, and Vietnam, with reports of epidemiological impact.^16^ ^(32-39)^ An intriguing pattern can be observed in Brazil: the neighbouring cities of Rio de Janeiro and Niteroi, although separated each other by a bridge, seems to present a very different introgression profile and epidemiological outcome. In Niterói, *w*Mel mosquitoes were deployed to the full extension of the city limits, which the authors reported to be associated with a substantial reduction of 69% of dengue incidence rate compared to predefined control areas.^40^ The outcomes in Rio de Janeiro were more modest and are reported in two manuscripts, with a summary schematic view with the timeline of *w*Mel deployment and monitoring in Fig. 1. The first large-scale deployment in Rio de Janeiro was conducted between January 2017 and December 2019. Despite releasing 67 million *w*Mel *Ae. aegypti* mosquitoes during 2017-2019, an average of only 32% *w*Mel introgression level into the wild population was achieved, which the authors reported to be associated with a reduction of 38% and 10% in dengue and chikungunya notifications, respectively.^41^ A second monitoring period was carried out in a subset of the intervention area between August 2021 and December 2023. More than 50,000 mosquitoes were captured in 12 neighbourhoods of Rio de Janeiro, and 7,613 *Ae. aegypti* females were individually screened for *w*Mel presence. In May 2022, a significant decline in *w*Mel frequency was observed across all the 12 neighbourhoods.^42^ Authors hypothesised the drop in *w*Mel frequency was associated with the introduction of the larvicide Spinosad (Natular^TM^, Clarke Mosquito Control Products, St. Charles, USA), which disrupted both *w*Mel-infected and -uninfected populations but only the latter was able to recover after an initial population crash likely due to fitness costs of *w*Mel on *Ae. aegypti* mosquitoes.^29^ ^30^ ^43^ In this article, we aim to update *Wolbachia* introgression in Rio de Janeiro, 30 months after *Ae aegypti* population crashed in the city, and 20 months after the last observation.^42^ By doing so, we plan to provide a snapshot of the current bacteria introgression status in Rio de Janeiro, shedding light on the long-term sustainability of this strategy to mitigate dengue transmission in endemic settings.

## MATERIALS AND METHODS

*Study area* - Field collections of adult mosquitoes were conducted in the same neighbourhoods of Rio de Janeiro as in the longitudinal sampling performed between August 2021 and March 2023.^42^ The total area covers 12 urban neighbourhoods: six of them received previous *w*Mel deployment between 2017-2019 from the World Mosquito Program (hereafter named as neighbourhoods A-F) and other six did not (named as G-L) (Fig. 2). This region covers an area of 33.5 km² (38.6% of the WMP release area) where approximately 495,000 people live (55.6% of the population under *w*Mel intervention), encompassing some of the most neglected communities in Rio due to urban violence.

**Fig. 2:**
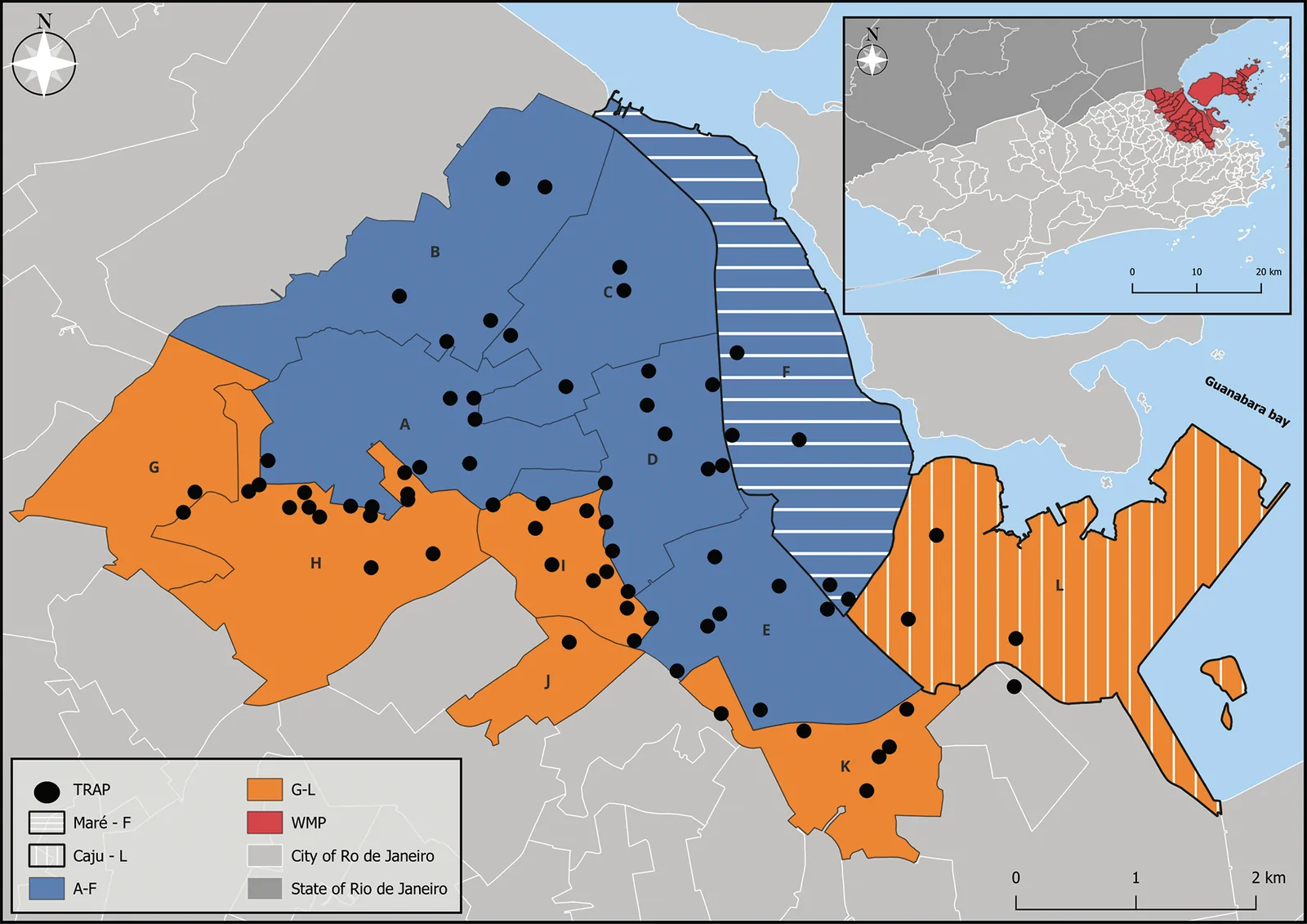
map of the city of Rio de Janeiro showing in red the total area where mosquito releases were conducted by the World Mosquito Program (WMP). Neighbourhoods included in the study are highlighted in blue (release areas; A: Complexo do Alemão; B: Olaria; C: Ramos; D: Bonsucesso; E: Manguinhos; F: Maré) and orange (non-release areas: G: Engenho da Rainha; H: Inhaúma; I: Higienópolis; J: Maria da Graça; K: Benfica; L: Caju). BG-Sentinel trap locations are marked with black dots. The neighbourhoods of Maré and Caju are indicated with horizontal and vertical hatching, respectively.

*Mosquito sampling* - Between November-December 2024, we installed 75 BG-Sentinel traps in the same 12 neighbourhoods and dwellings sampled in Pavan et al.^42^ Mosquito sampling was conducted in the same dwellings as Pavan et al.^42^ to avoid any bias by comparing *Wolbachia* frequency on different periods. We investigated whether any changes in *w*Mel frequency occurred 30 months after the crash in *Ae. aegypti* populations were observed. The traps were visited weekly until a predefined number of five *Ae. aegypti* females per trap were obtained. Captured mosquitoes were morphologically identified through taxonomic keys^44^ and individually screened for the presence of *Wolbachia w*Mel and relative density.

*In silico design and validation of oligonucleotides (primers) sets* - *Wolbachia* density was estimated through quantitative polymerase chain reaction (qPCR) using specific primers that were designed in this study for SYBR Green-based real-time PCR and amplify 115-bp of the *Wolbachia* surface protein gene (*wsp*; GenBank gene ID CP090948) of *w*Mel and 106-bp of the *Ae. aegypti* ribosomal protein S6 gene (*rps6*; GenBank gene ID 5563590). Briefly, genomic sequences were obtained from the Bacterial and Viral Bioinformatics Resource Centre v. 3.49 (https://www.bv-brc.org/) and NCBI (https://ncbi.nlm.nih.gov/) databases and sequences were aligned with the MultAlin tool available at http://multalin.toulouse.inra.fr/multalin/. We used *wsp* gene sequences of different *Wolbachia* strains (*w*Mel, *w*AlbA and *w*AlbB) aiming to select a region that would allow specific amplification of the *w*Mel strain. In the case of the *rps* gene, primers were designed in this highly conserved region of *Ae. aegypti*. Primer design was conducted in silico using an online tool (https://primer3.ut.ee/) to generate nucleotide pairs flanking target regions ranging from 50 to 150 base pairs. Finally, the quality of the designed oligonucleotides was evaluated using an online platform specifically developed for this purpose (https://www.idtdna.com). Primers were designed based on key thermodynamic parameters to ensure the specificity and efficiency of qPCR amplification. We selected primers with melting temperatures (Tm) above 45ºC and the difference between the Tm of forward and reverse primers lower than 5ºC. Additionally, the Gibbs free energy (ΔG) was calculated to assess the propensity for the formation of stable secondary structures, which the selected nucleotides had ΔG > -3 kcal/mol and ΔG > -10 kcal/mol to form hairpins and dimers.

The primers originally described by Ross et al.^45^ for *rps* presented a very low ΔG, favouring the formation of dimers. The new primers for *rps* are entirely located in the third exon of the *RpS6* gene, ensuring that amplification occurs exclusively in the transcribed regions, increasing efficiency and specificity. Comparative analyses indicate that the *RpS6* gene is highly conserved between *Ae. aegypti* and *Ae. albopictus*, which suggests that the same primers may be applicable to both species. Regarding the primer pairs for *w*Mel, the forward primer was designed in a region highly conserved among the *w*Mel, *w*AlbA and *w*AlbB strains, presenting only three specific nucleotides that differentiate *w*Mel from *w*AlbA. On the other hand, the reverse primer demonstrates greater specificity for *w*Mel, containing ten nucleotides unique to this strain and eight nucleotides shared between *w*Mel and *w*AlbA. Detailed information regarding the designed oligonucleotides is provided in Table.

**TABLE:** Overview of the specific oligonucleotides data. Details of designed oligonucleotides, including their names, accession IDs, genomic positions, target organisms, and nucleotide sequences

Gene target	GenBank accession (gene ID)	Genome	Organism	Oligonucleotides	Temperature de melting (Tm) (**º**C)	Position (nt)
rpS6	5563590	AaeL_AAEL000032	*Ae. aegypti*	aedesRpS6_F_1 (5'-GTGTCCGTCTGCTGCTGAAGA -3') aedesRpS6_R_1 (5'-GACAGGTTCTGGTCAACGATGC -3')	F: 59.5ºC R: 58.2ºC	Primer forward-9005-9025 Primer reverse-9088-9110
wsp	CP090948	546165.19 / *Wolbachia* endosymbiont of *Aedes aegypti w*Mel_GV_13	*w*Mel, *w*AlbA, *w*AlbB	wmelwAlbAwsp_F_1 (5'-ATGTAACTCCAGAAGTCAAACTTTATG -3') wmelwsp_R_2 (5'- GCATCAGCAGCCTGTCCG-3')	F: 53.5ºC R: 59.3ºC	Primer forward- 1023582-1023609 Primer reverse- 1023679-1023697

In grey: exon; green: regions conserved among *w*Mel, *w*AlbA, and *w*AlbB; in blue: regions shared between *w*Mel and *w*AlbA; in yellow: regions unique to *w*Mel.

Laboratory-reared *Ae. aegypti* mosquitoes infected with *w*Mel (F5 generation from Tubiacanga, Rio de Janeiro, Brazil) and not infected with *w*Mel (Paea strain) were used to test primer efficiency and specificity. Mosquitoes were reared in an incubator (Solab model SL-200/364) from egg to adult stage, under a photoperiod of 12 h of light and dark (LD 12:12) and at 28^o^C. Females aged 0 to 3 days were flash-frozen in liquid nitrogen. Subsequently, each individual body was homogenised using a pestle, and the resulting material was stored in 350 μL of Buffer RLT Plus (reagent of the Allprep DNA/RNA Mini Kit extraction kit, Qiagen, Hilden, Germany) in a -80ºC freezer until subsequent extraction of genetic material. Total DNA and RNA were extracted using the Allprep DNA/RNA Mini Kit (Qiagen, Hilden, Germany), according to the manufacturer's protocol and the DNA was used in the primer testing assays. The concentration of the extracted DNA was determined using the Qubit 4 Fluorometer quantification system (Thermo Fisher Scientific, Waltham, USA). A pool of ten individuals (each individual sample initially had ~10 ng/µL of DNA) of each positive (*w*Mel-infected) and negative (*Wolbachia*-free mosquitoes) control was used to calculate oligonucleotide efficiency and specificity through serial dilutions ranging from 1/1x to 1/32x (12.5-400-ng total DNA). The experiments were performed in a QuantStudio 6, using 7.5 µL of Power SYBR Green PCR Master Mix (Thermo Fisher Scientific), with 0.75 µL of each oligonucleotide at 10 mM, 4 µL of DNA, and 2 µL of water in the QuantStudio^TM^ 6 Flex Real-Time PCR System (Applied Biosystems, Waltham, MA, USA), under the following regime: an initial incubation at 50ºC for 2 min, followed by an initial denaturation at 95ºC for 10 min. The amplification protocol consisted of 40 cycles of denaturation at 95ºC for 15 s and annealing/extension at 60ºC for 1 min. A melt curve analysis was subsequently performed under the following conditions: 95ºC for 15 s, 60ºC for 1 min, and a gradual increase to 95ºC for 15 s. All reactions were done with three independent replicates.

*DNA extraction and Wolbachia quantification in field sampled mosquitoes* - Individual females of *Ae. aegypti* were massed in L15 culture medium (Leibovitz) in a Tissue Lyser II apparatus (Qiagen, Hilden, Germany) for 60 s at 50 Hz and the DNA was extracted using the NucleoSpin^®^Tissue extraction kit (Macherey-Nagel, Düren, Germany) following the manufacturer's protocol. The DNA was quantified in a Qubit 4 Fluorometer quantification system (Thermo Fisher Scientific, Waltham, USA). The relative quantification of *w*Mel in whole mosquitoes was performed with quantitative PCR by amplifying the *Wolbachia wsp* gene, using the *rps6* of *Ae. aegypti* as an endogenous control.^46^ Quantitative PCR was performed following the same parameters, reagents, equipment and were run under the same cycling conditions as previously outlined for the primers test. The highest resulting delta threshold cycle (ΔCt) was used as a reference sample for delta delta Ct calculations (ΔΔCt).^47^

*Statistical analysis* - Descriptive analysis considered three time points to represent the spatiotemporal fluctuation of *w*Mel in Rio de Janeiro. The first two time points used the raw data available in Pavan et al.^42^ We selected November/2021 to represent the beginning of the mosquito season in the city and when *w*Mel frequency peaked. We selected July/2022, a few months after the population crashed (May/2022), and sampling was restored to similar numbers as to before the crash. The last time point represents the most recent sampling, in Nov/2024, 30 months after the population crash.

The relative frequency of *w*Mel was compared after the classification of neighbourhoods according to the presence/absence of previous *Wolbachia* deployments. We fitted a generalised linear model (GLM) with a negative binomial distribution with the number of *Wolbachia*-infected mosquitoes per trap as the depended variable and the occurrence of releases as the explanatory variable. In addition, we included a log-transformed offset of the total number of mosquitoes tested pert trap, allowing for the interpretation of the model coefficients in terms of relative rates. Model fit was examined by checking heteroscedasticity, residuals dispersion and the presence of outliers with the R package DHARMa.^48^ To test the significance of the predictor variable, we used the type II analysis of deviance. These analyses were performed in the R environment (version 3.6.2).^49^

Assuming *Wolbachia*-mediated arbovirus blocking effect is often density dependent^50^ we also quantified the endosymbiont density relatively to the *rps6* of *Ae. aegypti* in field-caught mosquitoes. The *w*Mel density was compared between females captured in areas that received or not *Wolbachia* deployments, as well as with a Fiocruz lab colony using the Kruskal-Wallis test, followed by Dunn multiple comparisons post hoc tests.

*Geographic information and choropleth maps* - We recorded and stored all entomologic field-derived data, such as the number of trapped and tested adult mosquitoes, along with geocoded address of traps and the neighbourhood, and the *Wolbachia* screening status per sample, in a single database.^25^ The maps were produced in Quantum GIS version 3.10.2 (QGIS, https://www.qgis.org). We created choropleth maps to help visualise the *Wolbachia* frequency in sampled *Ae. aegypti* mosquito population among the 12 areas of the city. We built the choropleth maps by dividing the *w*Mel frequencies in five intervals: 0.1-20%; 20.1-40%; 40.1-60%; 60.1-80%; 80.1%-100%.

## RESULTS

*Primer evaluation* - Melting curve analysis revealed a single peak for the *wsp* gene, suggesting that only one fragment is amplified per reaction (Fig. 3). The reaction efficiency for the oligonucleotides targeting the *wsp* gene of *w*Mel falls within the optimal range, with 92.3% efficiency. Regarding the *Ae. aegypti rps6* gene, amplification occurred in both *w*Mel-infected and *Wolbachia*-free mosquitoes (Fig. 4), and the melting curve also presented a single peak in both mosquito groups. The efficiency of the primers was 103.0%, also falling into the optimal range.

**Fig. 3:**
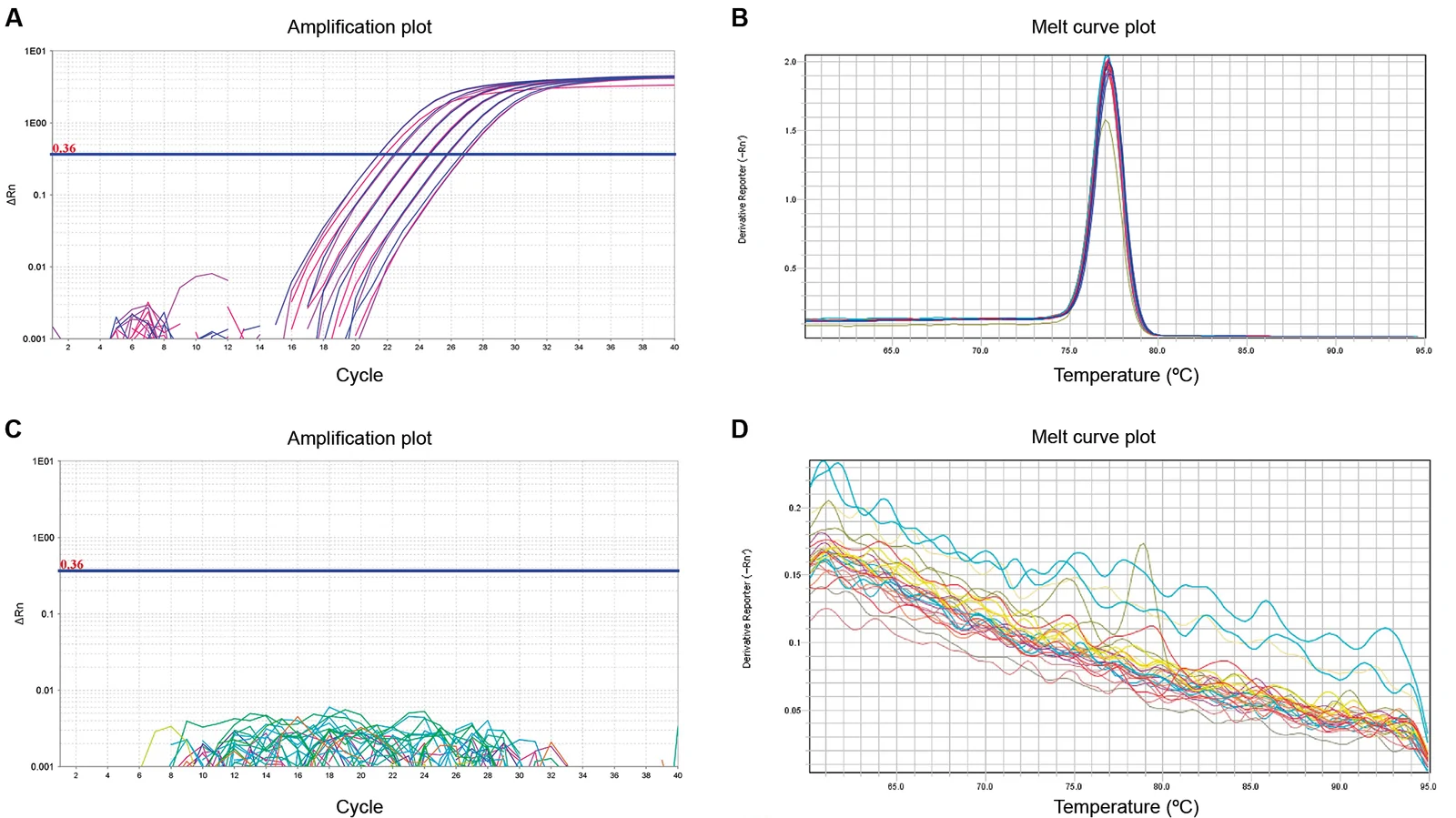
oligonucleotides for the *wsp* gene of *w*Mel - (A) Amplification curve for the *wsp* gene of *Wolbachia w*Mel, using DNA from *Aedes aegypti* females with *w*Mel; (B) Melting curve for the *wsp* gene of *Wolbachia w*Mel, using DNA from *Ae. aegypti* females infected with *w*Mel; (C) Amplification curve for the *wsp* gene of *w*Mel, using DNA from *Wolbachia*-free *Ae. aegypti* females (*PAEA* strain); (D) Melting curve for the *wsp* gene of *Wolbachia w*Mel, using DNA from *Wolbachia*-free *Ae. aegypti* females (*PAEA* strain).

**Fig. 4:**
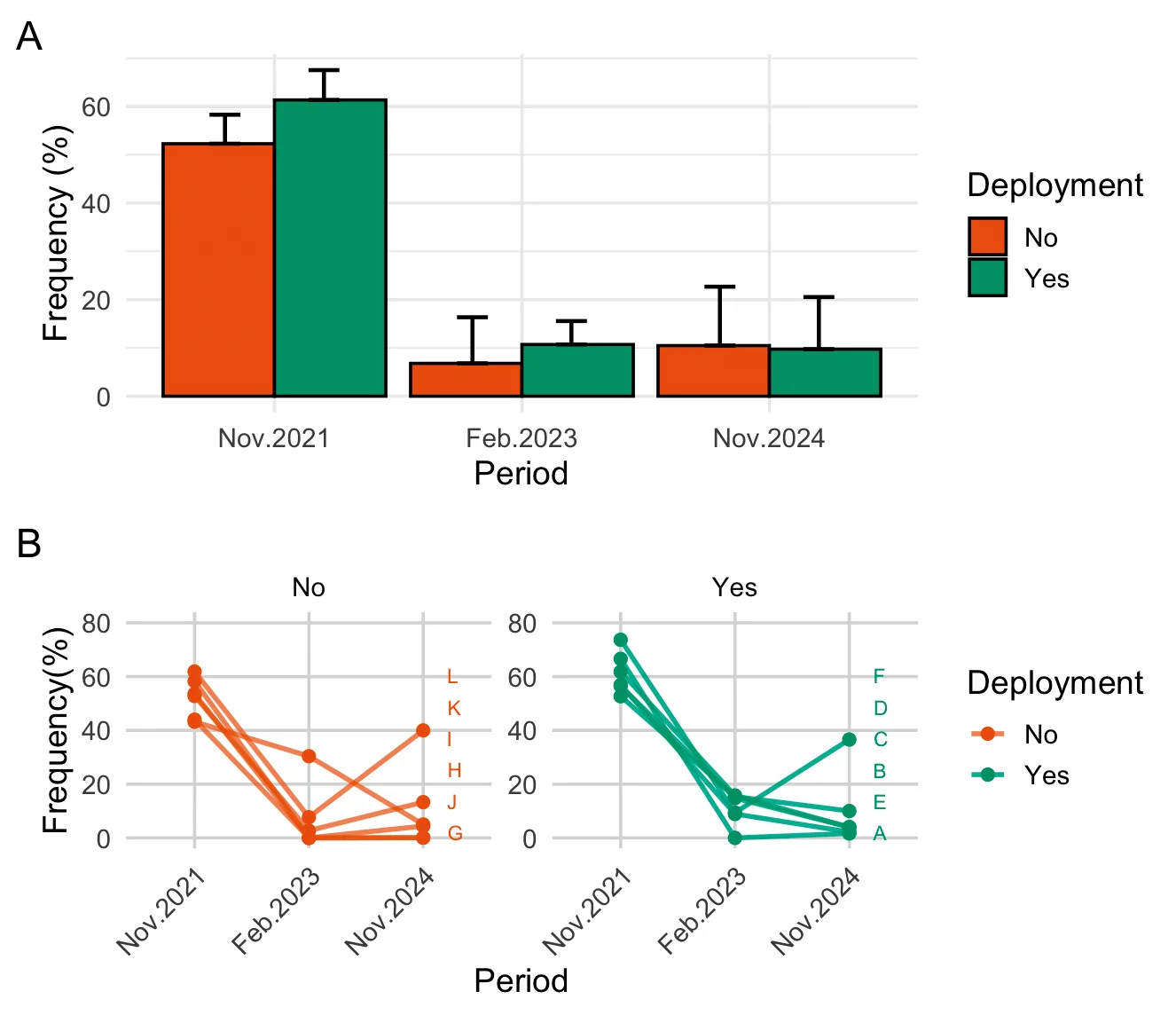
frequency of mosquitoes (out of five) detected with the *w*Mel strain of *Wolbachia* per trap in neighbourhoods with and without previous mosquito releases by the World Mosquito Program (WMP).

*Frequency of Wolbachia wMel in field Ae. aegypti* - In total, we tested 375 *Ae. aegypti* females from 12 neighbourhoods over the period in which traps remained in the field. There were no significant differences on the number of *Wolbachia*-infected mosquitoes according to the occurrence of previous *Wolbachia* deployments. The coefficient for this explanatory variable was negative but not statistically significant (estimate = -0.3514, z = -0.752, p = 0.452). This finding was supported by the Type II analysis of deviance (χ² = 0.568, df = 1, p = 0.451), indicating a similar frequency of *w*Mel mosquitoes in areas adjacent to release zones (Fig. 4), as previously reported (Pavan et al.^42^).

The frequency of *w*Mel in release and non-release areas did not change much between the period of the population crash and the subsequent collection. In March 2023, when the monitoring in Pavan et al. ended,^42^ the *Wolbachia* frequencies in release and non-released areas were 13.2% and 5.01%, respectively. In November 2024, the frequency of *w*Mel in released areas dropped to 8%, whereas in the non-release areas it increased to 12% (Fig. 5A). Additional information regarding *w*Mel frequency is available if we aggregate data by neighbourhood. Considering the neighbourhoods with previous releases (A-F), the *w*Mel frequency was reduced between March 2003 and November 2024, with the exception of the area F. Regarding the neighbourhoods in which no *w*Mel deployment was carried out, in the areas G and J no *Wolbachia*-infected mosquitoes were captured in both evaluation periods. However, a significant increase in *w*Mel frequency was observed in areas K and L, both located in the southern limit of our study area (Fig. 5B). Remarkably, four neighbourhoods had no *w*Mel mosquitoes captured in the BG-Sentinel traps by the end of March 2023. Of these, one had previous releases (A), and three others were without previous deployments (G, H, J). On November 2024, the A and H sites had a frequency of 1.54% and 4.44% of *w*Mel-infected mosquitoes, respectively, whereas sites G and J remained without *w*Mel-infected mosquitoes (Fig. 5B).

**Fig. 5:**
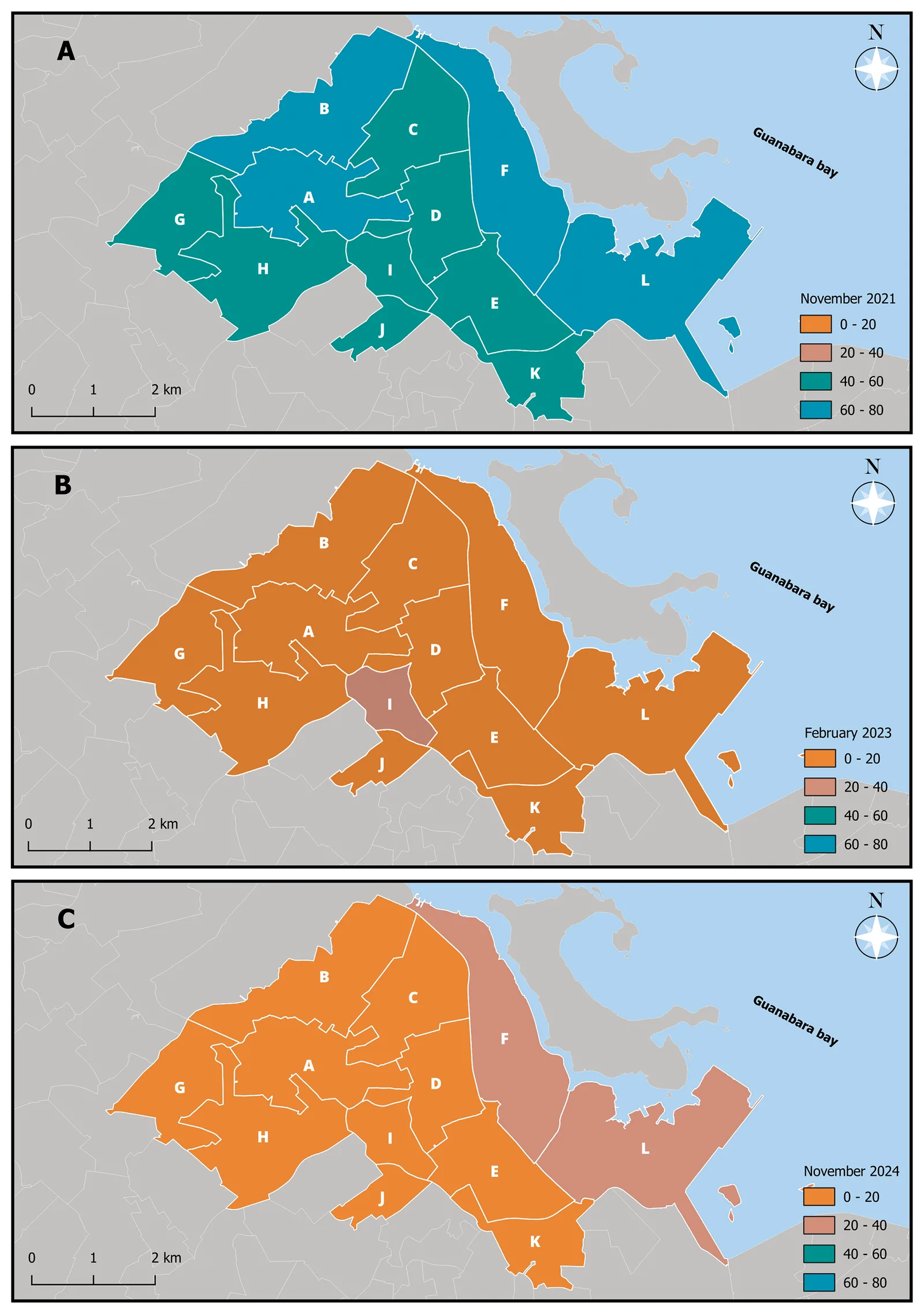
frequency of *Wolbachia* in female *Aedes aegypti* from the field covering a total period of 3 years, using the raw data from Pavan et al.^42^ at November 2021 and July 2022. In light blue (2023 data) and blue (2024 data) are the average frequencies of *w*Mel in neighbourhoods which received *Wolbachia* deployments; in light orange (2023 data) and orange (2024 data) are the frequencies of *w*Mel in areas with no previous *Wolbachia* releases. (A) *w*Mel frequency of neighbourhoods with and without previous releases. (B) Data presented per neighbourhood. Notably, Engenho da Rainha and Maria da Graça had no *w*Mel-infected *Ae. aegypti* captured in both periods.

**Fig. 6:**
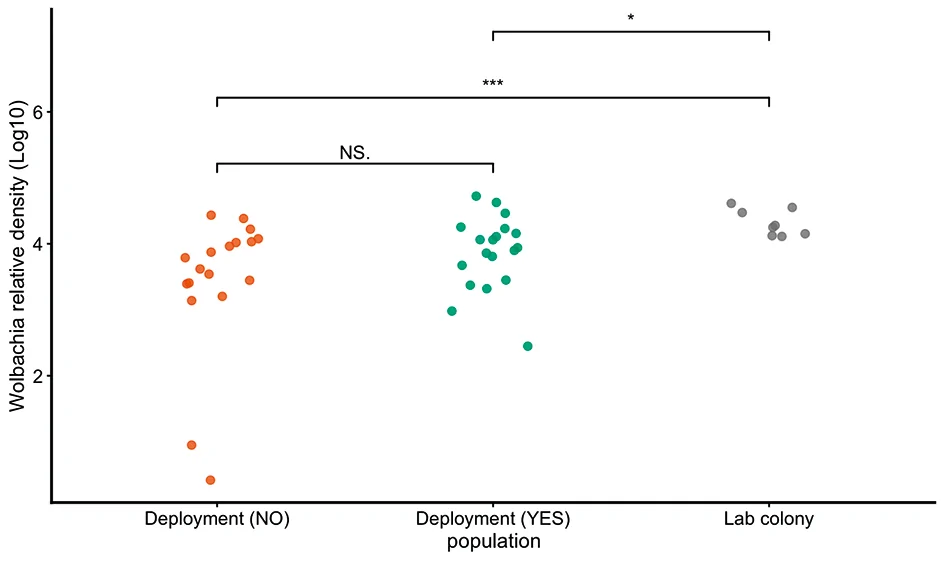
relative density of *Wolbachia* in *Aedes aegypti* females from the *w*Mel colony and from the field, captured in BG-Sentinel traps installed in areas with or without previous *Wolbachia* releases. The *wsp* gene was used as a target and the *rps6* gene as an endogenous normaliser. Each symbol represents one single mosquito female.

The density of the *w*Mel strain in *Ae. aegypti* mosquitoes was consistent between those individuals caught in areas with or without previous deployment, but those from the lab colony had a higher *Wolbachia* density than the ones collected in neighbourhoods that had no releases (H = 11.23, df = 2, p = 0.003, followed by Dunn: Z = 18.28, p < 0.05)(Fig. 6).

## DISCUSSION

The deployment of *Ae. aegypti* carrying *W. pipientis* to mitigate dengue transmission has been underway in different epidemiological settings. In Australia, the *w*Mel strain of *Wolbachia* successfully established in local *Ae. aegypti* populations, reaching fixation and maintaining high prevalence accompanied by a 96% reduction in dengue incidence.^18^ ^51^ Similarly, in Yogyakarta, areas achieving > 70% *Wolbachia* prevalence reported a 77% reduction in dengue cases and an 86.2% decline in dengue-related hospitalisations.^52^ The deployment of *w*AlbB in Malaysia resulted in an initial average 40% reduction of dengue cases in the intervention sites, rising to > 60% afterwards.^34^ ^35^ ^53^ The release of *w*Mel strain in Brazil had divergent patterns in the neighbouring cities of Rio de Janeiro and Niterói for reasons that remain unclear. In Niterói, despite heterogeneous introgression patterns across landscapes, an overall 69% reduction in dengue transmission was obtained,^40^ while in Rio de Janeiro, a fluctuating *w*Mel prevalence around 32% led to modest reductions in dengue (38%) and chikungunya (10%) incidence.^41^ Considering the epidemiological outcome of *Wolbachia* seems to be tightly connected with the level of introgression achieved and the maintenance of high frequency over time, the spatiotemporal evaluation of *Wolbachia* is of utmost relevance to determine its success.

The World Mosquito Program released 67 million *Ae. aegypti* mosquitoes to cover approximately 6.9% of Rio de Janeiro city's area between August 2017 and December 2019.^41^ From August 2021 to March 2023 an independent monitoring was able to follow significant changes in *w*Mel frequency.^42^ The *w*Mel frequency remained relatively stable between 30-40% until June 2022, when a sharp decline in both *w*Mel-infected and uninfected *Ae. aegypti* populations was observed in Rio de Janeiro. This coincided with the introduction of the new larvicide Spinosad, to which mosquitoes showed full susceptibility. Most likely, the fitness cost associated with this *Wolbachia* strain on mosquito life-history traits prevented its recovery after the Spinosad application coverage was reduced by the local public health office.^28^ ^30^ ^31^ ^42^ ^(54-57)^ Our monitoring revealed that in March 2023 only ~10% of field-caught mosquitoes had *w*Mel*.* In the present study, we investigated the current profile of *w*Mel introgression in Rio de Janeiro to evaluate potential changes in *Wolbachia* dynamics after its collapse as reported elsewhere.^42^ *Wolbachia* frequency remained at low levels similarly to those observed in Pavan et al.^42^ The persistence of *w*Mel frequency at ~10% suggest its stabilisation over the last couple of years in Rio de Janeiro. Considering the reproductive advantage conferred by *Wolbachia* is frequency-dependent, its persistence at the low frequencies reported herein will likely be insufficient to trigger a further increase without needing additional releases. Most importantly, there are concerns whether *Wolbachia* frequency at this level can still be effective for protecting against dengue outbreaks.^15^ ^58^

The introduction of the *w*Mel strain of *Wolbachia* into native *Ae. aegypti* populations has been proposed as a promising and sustainable strategy for arbovirus control, based on the assumption that, once a high prevalence threshold is surpassed, *Wolbachia* frequencies remain stable over time.^59^ ^60^ ^61^ However, the long-term persistence of *Wolbachia* in field populations appears to be highly dependent on a range of ecological and anthropogenic pressures, including insecticide exposure and climatic variability. In Brazil, the initial field release of *w*Mel-infected *Ae. aegypti* occurred in the geographically isolated fishing community of Tubiacanga, Rio de Janeiro. Although early invasion dynamics were promising, *Wolbachia* frequencies declined sharply following the cessation of releases, coinciding with intensive household level applications of pyrethroid-based adulticides.^16^ ^62^ A more extensive collapse in *w*Mel prevalence was observed across a broader urban area, likely associated with the expanded use of the larvicide Spinosad.^41^ Similar patterns of instability have been documented elsewhere; for example, in Tri Nguyen village, Vietnam, *w*Mel frequencies, which had surpassed 90% during cooler periods, dropped to approximately 24% during the hot-dry season, suggesting a pronounced sensitivity to seasonal thermal stress.^20^ In Rio de Janeiro, seasonal oscillations in *Wolbachia* prevalence have likewise been recorded, with the lowest frequencies observed during the summer months — coinciding with the peak incidence of dengue transmission.^41^ As in Tri Nguyen village, results in Rio de Janeiro also showed small-scale heterogeneity, but most likely due to additional releases conducted by the WMP.^63^ The observation that only the neighbourhoods of Maré (area F) and Caju (area L) exhibited an increase in *w*Mel frequency is likely attributable to successive rounds of mosquito releases, as reported in local media sources.^63^ The necessity of supplementary releases in areas where *Wolbachia* frequencies remain below optimal thresholds should be considered an integral and ongoing component of vector control programs, particularly in light of the environmental factors that can influence *w*Mel dynamics. Additional local boost releases to overcome micro-geographic drops on *Wolbachia* frequency must be done when routine surveillance detects poor introgression in areas where deployment has taken place. Furthermore, given the historical role of Rio de Janeiro as a major port of entry for both dengue serotypes and genotypes, ensuring that *w*Mel frequencies remain at protective levels in previously targeted areas should take precedence over the expansion of releases into new localities.^64^ ^65^ The prevalence threshold of *w*Mel to effectively protect local communities is still unknown, but it must consider the *Wolbachia* strain, the spatiotemporal heterogeneity of its frequency, herd immunity, and dengue serotype, to mention a few. A preliminary result showed that introgression < 45% was not associated with a reduced dengue incidence during 2024 in Rio de Janeiro city.^66^ However, further investigations are required to understand whether this threshold can be extrapolated elsewhere.

An additional consequence of suboptimal *w*Mel frequencies in the field is the reduced level of community protection, as lower prevalence of *Wolbachia* is expected to result in diminished interruption of arbovirus transmission. The epidemiological consequences of *Wolbachia* deployment are unlikely to be uniform across transmission settings and should be interpreted in light of local arbovirus dynamics. Rather than fully blocking dengue transmission, the introduction of the *w*Mel strain is generally expected to reduce the effective reproductive number (R₀). Modelling studies suggest that when transmission intensity is low to moderate, such reductions may be sufficient to interrupt transmission. However, in highly endemic contexts the same effect may translate primarily into decreases in incidence rather than complete suppression of outbreaks.^67^ This distinction is particularly relevant for settings such as Rio de Janeiro, where dengue transmission can reach very high levels during epidemic years. Notably, in 2024, Rio de Janeiro experienced its most severe dengue outbreak in the past decade.^68^ Clearly, the reported increase in dengue transmission is not directly related to the drop in *Wolbachia* frequency given it covers less than 7% of the city's total area. However, spatiotemporal Bayesian models were fit to estimate the effect of introgression on dengue incidence during 2020-2024.^67^ Authors observed the *Wolbachia* deployment was not associated with reductions in dengue incidence in 2024, amid a large dengue epidemic was reported in Rio de Janeiro city.^67^ Moreover, introgression < 45% was not associated with reduced dengue incidence.^67^ The updated frequency of *w*Mel in Rio de Janeiro revealed all 12 neighbourhoods sampled had introgression levels below the 45% threshold suggested.^67^

The *w*Mel density of field-caught mosquitoes from the area without previous releases was significantly lower than the lab colony, following the same pattern as observed before.^43^ As extensively reported, high temperatures can negatively impact *w*Mel density in *Ae. aegypti*.^23^ ^26^ ^27^ Considering lab mosquitoes are reared in optimum insectary conditions, with a stable temperature of 27 ± 2ºC, *Wolbachia* density of lab colony mosquitoes is expected to be high. Regarding arbovirus mitigation, it is well known that the pathogen-blocking phenotype associated with *Wolbachia* depends on its density.^69^ ^70^ ^71^ Therefore, seasonal fluctuations in *w*Mel density may affect *w*Mel effectiveness, especially if a decrease is observed during the warmer periods in which dengue transmission is naturally more intense.

This study has limitations. The sampling strategy encompassed only a subset of the *w*Mel deployment area, thereby constraining the ability to generalise findings to non-monitored regions. Additionally, BG-Sentinel traps were preferentially installed in the same households as those used in Pavan et al.,^42^ which facilitates temporal comparison but may perpetuate any sampling biases present in the original dataset. Despite these constraints, the study offers a robust and independent assessment of the spatiotemporal distribution of the *w*Mel strain in Rio de Janeiro. These findings highlight the critical importance of adopting an Integrated Vector Management (IVM) framework to enhance the efficiency and sustainability of vector control efforts and disease prevention strategies and to engage in ongoing monitoring and adaptative releases when deploying *Wolbachia*.

## Data Availability

After publication the data will be available on demand to corresponding author.
