# Supplementary material for: Wolbachia introgression in Rio de Janeiro remains at sub-optimal levels 30 months after its decline: challenges in the sustainability of wMel interventions for dengue control
Source: Mem Inst Oswaldo Cruz. 2026 Oct 5;121(Suppl 1):e250212. doi: 10.1590/0074-02760250212 (PMC13645213; doi:10.1590/0074-02760250212)
Supplement: Peer review history

# Reviewer report: FIRST REVIEW ROUND - REVIEWERS' COMMENTS

## REVIEWER #1

Overall Assessment

This study examines the persistence of Wolbachia wMel in Aedes aegypti in Rio de Janeiro 30 months after a major population collapse. The topic is highly relevant to dengue control, and the longitudinal monitoring effort is commendable. However, the manuscript is difficult to follow in places, lacks key data presentation (notably spatial maps), and makes confident claims that are not always well supported by evidence. Methodological choices, particularly the introduction of a new molecular assay without proper validation, further complicate interpretation. Substantial revision is needed before the study can be considered for publication

Major Comments

1) Spatial presentation of results

• The study is explicitly about neighborhood-level heterogeneity in wMel persistence, yet spatial information is only presented in tables and bar charts. Without maps or spatial analyses, it is very hard for the reader to discern geographic patterns or continuity of areas with low prevalence.

• Spatial mapping would considerably strengthen the manuscript and should be included. At present, the narrative feels fragmented and unconvincing.

2) New molecular assay and comparability over time

• A new TaqMan qPCR assay was developed, justified only by a claim that the prior assay could form primer dimers. However, no validation data are provided (e.g. sensitivity, specificity, reproducibility, or comparison with the established method).

• Because temporal comparisons are central to the manuscript, using different assays across years without cross-validation undermines confidence in the claim of declining wMel prevalence.

• At minimum, authors should present validation data or side-by-side comparisons to demonstrate assay equivalence. Without this, their conclusions risk being artefactual.

3) Sample size and statistical robustness

• The study tested only 375 females across 12 neighborhoods (~39% of the release area?). This is a limited dataset for such strong claims.

• Please provide confidence intervals around prevalence estimates and clarify the statistical power.

• The GLM model is mentioned but not well described (covariates, diagnostics, rationale for negative binomial). Supplementary materials should include model diagnostics.

4) Attribution of Wolbachia loss to Spinosad

• The manuscript strongly attributes the decline in wMel to spinosad larvicide use. While plausible, this is a correlative association. Other drivers (climate variability, immigration of uninfected mosquitoes, stochastic drift) are not fully considered. The discussion should be more balanced and avoid over-interpretation.

5) Interpretation of epidemiological impact

• The link to Rio's 2024 dengue epidemic is tenuous, as Wolbachia releases covered <7% of the city. The text should avoid implying causation at this scale. The current framing is overly confident and risks overstating public health impact.

6) Figures and clarity

• Figure 5: Barplots of Wolbachia prevalence lack confidence intervals. Given small sample sizes, CIs (e.g., binomial or Wilson) are essential to show uncertainty. Without them, differences may appear more robust than they are.

• I assume that in Figure 6: "NO Wolbachia" means individuals without Wolbachia infection. Yet, this group is shown with positive values for the loads. This is confusing. The authors must clarify how infection status was defined, why density values are shown for negatives, and whether these represent assay noise, sub-threshold infections, or plotting artefacts.

• qPCR output plots currently in the main figures could be moved to Supplementary Data, with only summary results retained. This would streamline the main text.

7) Programmatic implications

• The conclusion that Wolbachia must be "reinforced before expanding elsewhere" is sensible, but currently too general. What prevalence threshold is considered protective? Explicitly linking findings to WHO guidelines or programmatic targets would increase the utility of the recommendations.

Minor Comments

1) Abstract: rephrase "Regardless the wMel infection status" → "Regardless of the wMel infection status."

2) Introduction: More clearly connect the cited heterogeneity studies (refs 16-20) to Rio's unique context.

3) Methods: Provide more detail on neighborhood selection and improve Figure 1 map.

4) Results: Present neighborhoods with zero wMel in tabular form for clarity.

5) Discussion: Expand the point on seasonal thermal stress and its likely contribution to Wolbachia instability in Rio.

Recommendation: Major revision.

The study is potentially valuable but is currently weakened by limited spatial presentation, lack of molecular assay validation, and overconfident causal claims. Addressing these issues with additional data, more cautious interpretation, and clearer figures will be necessary for publication.

## REVIEWER #2

This paper examining Wolbachia establishment in Rio de Janeiro for dengue control is a little confusing in its framing and the conclusions it draws from very limited data. The general narrative is that Wolbachia has somehow been established and then lost from mosquito populations in Rio and this is somehow connected to the ecological perturbation of using Spinosad insecticides. Everything is presented as correlation without demonstration of causality. I think a much simpler explanation for the observed data is that Wolbachia establishment never succeeded in Rio initially. Successful introgression requires the Wolbachia frequency to exceed the unstable point as defined by the models of Michael Turelli. The reported Wolbachia frequencies in Rio -- around 30-40% are very close to the measured unstable point of approx. 0.3 as measured in Cairns, Australia for the wMel strain. Given this closeness I think a more likely and simpler explanation than invasion followed by Wolbachia loss is that invasion was never accomplished (as defined by robustly exceeding the unstable point). Turelli's models predict that the closer the Wolbachia frequency is to the unstable point the slower the introgression dynamics will be -- either for invasion or loss and indeed what appears to be happening in Rio is a gradual loss of infection, consistent with the modelling, and this loss is happening slowly as predicted. The issue of the Spinosad insecticide application having some causal link with this loss seems highly unlikely as both populations of Wolbachia infected and uninfected mosquitoes are expected to be equally susceptible to the insecticide and so it should not be important in influencing the introgression dynamics. So, in my opinion the authors are trying to weave a complex narrative around something that is much simpler, and that is that Wolbachia was never successfully established and is slowly decaying in frequency in these areas. There is no need to invoke more complicated explanations and in fact this could be quite misleading. The key point as mentioned by authors is that in adjoining neighbourhoods in Niteroi, the introgression is complete and stable and experiences the same weather conditions as Rio, arguing against a general climate issue affecting introgression. Interestingly, when releases were first undertaken in Jurajuba, in Niteroi, there was initial difficulty in establishing Wolbachia, requiring re-releases but once established it was extremely robust.

So, while the paper attempts to make the claim that complex environments might lead to loss of Wolbachia a better conclusion in my opinion is that complex environments may make establishment of Wolbachia more challenging. Indeed, in this setting there were poorer neighbourhoods that routinely experience elevated levels of violence, interrupting release activities for weeks at a time during deployment. These disruptions together with possible underdosing may be much more likely to explain the introgression dynamics. While initial dose can be an important consideration, we also need to consider the role of the unhatched egg bank in certain settings which will influence establishment dynamics and could be an important but relatively unexplored critical element of the mosquito ecology that will influence Wolbachia establishment. Indeed, it may have been a critical element explaining the dynamics of Wolbachia in Jurajuba, Niteroi. Considering the novelty of the intervention I think it is normal that as we undertake deployments at increasing scale in a Public Health context we can expect some failures and optimisation that will be required in learning the best ways to deploy. However, that is very different from thinking that the method is inherently unstable. The success in Niteroi is evidence against the latter conclusion.

## AUTHORS' RESPONSE TO THE REVIEWERS

REVIEWER COMMENTS:

Reviewer: 1

Overall Assessment

This study examines the persistence of Wolbachia wMel in Aedes aegypti in Rio de Janeiro 30 months after a major population collapse. The topic is highly relevant to dengue control, and the longitudinal monitoring effort is commendable. However, the manuscript is difficult to follow in places, lacks key data presentation (notably spatial maps), and makes confident claims that are not always well supported by evidence. Methodological choices, particularly the introduction of a new molecular assay without proper validation, further complicate interpretation. Substantial revision is needed before the study can be considered for publication.

**A:** We appreciate Reviewer observations, although we believe most of the key data requested is available in the publication in which we demonstrated the collapse of *Wolbachia* after individually screening by RT-qPCR wMel presence in 7,613 field-collected *Ae. aegypti* (Pavan et al. 2025). The aim of the manuscript under evaluation is to share an update of *w*Mel frequency exactly 30 months after it crashed due to an increase in coverage of the larvicide Spinosad in the study area.

We acknowledge the fact this manuscript is an update of the actual *Wolbachia* frequency was not properly described. Therefore, we edited several sections of the manuscript to make it clearer in the second version, and created a new Figure with a timeline of Rio de Janeiro large-scale deployments, from 2019-2025 (became Figure 1 in the manuscript, as it is below). The other points raised will be discussed in the following comments.

**Fig. 7:** 

Major Comments

1) Spatial presentation of results

• The study is explicitly about neighborhood-level heterogeneity in wMel persistence, yet spatial information is only presented in tables and bar charts. Without maps or spatial analyses, it is very hard for the reader to discern geographic patterns or continuity of areas with low prevalence.

• Spatial mapping would considerably strengthen the manuscript and should be included. At present, the narrative feels fragmented and unconvincing.

**A:** We appreciate Reviewer observation regarding the spatial information of *w*Mel persistence. As mentioned now in the manuscript, we selected three representative periods of the time series from Aug/2021 to Dec/2024:

November 2021 to represent the beginning of the mosquito season in the city and when wMel frequency peaked.

July/2022, a few months after the population crashed in May/2022, and sampling was restored to similar numbers as to before the crash.

Nov/2024 The last time point represents the most recent sampling, exactly 30 months after the population crash.

We did choropleth maps to show the spatiotemporal variations on *Wolbachia* frequency during the timeframe of three full years, from November 2021-2024. The maps are now listed as Figure 5 and are presented below.

**Fig. 8:** 

2) New molecular assay and comparability over time

• A new TaqMan qPCR assay was developed, justified only by a claim that the prior assay could form primer dimers. However, no validation data are provided (e.g. sensitivity, specificity, reproducibility, or comparison with the established method).

• Because temporal comparisons are central to the manuscript, using different assays across years without cross-validation undermines confidence in the claim of declining wMel prevalence.

• At minimum, authors should present validation data or side-by-side comparisons to demonstrate assay equivalence. Without this, their conclusions risk being artefactual.

**A:** We appreciate the reviewer's concern, but *Wolbachia* was screened in Pavan et al. (2025) through conventional PCR. Therefore, since we made qPCR assays, this new method is more sensitive than the one used previously. This way, we probably detected *Wolbachia*-positive samples even when *Wolbachia* load was below the limit of detection of a conventional PCR.

We believe, however, that we could added more information on these new primer pairs. As previously explained in the Methods section, the new primers were validated in silico to ensure high specificity and efficiency of qPCR amplifications. Moreover, experimental validation steps were performed, such as the calculation of primer efficiency, melting curve, and analysis of amplification plots of serial dilutions of samples. In this new version, we also added information on the limit of quantification of primer pairs, and calculations of coefficient of variation to determine their specificity and reproducibility (lines 220-226, and 294-298).

3) Sample size and statistical robustness

• The study tested only 375 females across 12 neighborhoods (~39% of the release area?). This is a limited dataset for such strong claims.

**A:** We believe our dataset is sufficient to provide an update of the Wolbachia frequency 30 months after its collapse in Rio de Janeiro, described in Pavan et al. (2025). Just to reinforce, in Pavan et al. (2025), the collapse was identified after establishing a longitudial sampling of 20 monthis in which 19,437 *Ae. aegypti* females were sampled and 7,613 individually screened for *Wolbachia*. In this manuscript, we returned to the same 75 collection points and updated the *w*Mel frequency after collecteg 5 females per trap, i.e., 375 individuals.

• Please provide confidence intervals around prevalence estimates and clarify the statistical power.

**A:** We have provided the confidence intervals over prevalence estimates.

• The GLM model is mentioned but not well described (covariates, diagnostics, rationale for negative binomial). Supplementary materials should include model diagnostics.

**A:** These analysis details were included in the methods section (lines 234-249), as well as model diagnosis plots are now available in Supplementary material.

4) Attribution of Wolbachia loss to spinosad

• The manuscript strongly attributes the decline in wMel to spinosad larvicide use. While plausible, this is a correlative association. Other drivers (climate variability, immigration of uninfected mosquitoes, stochastic drift) are not fully considered. The discussion should be more balanced and avoid over-interpretation.

**A:** We believe there is misunderstanding regarding a possible claim in this manuscript that *Wolbachia* loss was caused by Spinosade. We actually showed it in another paper (Pavan et al. 2025, ref #42 of the cited references). At Pavan et al. 2025, we conducted a 20-month longitudinal investigation and sampled 50,240 mosquitoes, i.e., the claim that Spinosade caused the *Wolbachia* loss was based on a much higher sampling effort. At this manuscript, we conducted a new round of mosquito sampling in the same 75 locations as reported in Pavan et al. (2025). I.e., the sampling was conducted over the same 12 neighborhoods to update the *w*Mel frequency at Rio de Janeiro 30 months after *Wolbachia* crashed (as explained in details at Pavan et al. 2025).

Still, we agree with the reviewer when he/she mentions that other drivers (climate variability, immigration of uninfected mosquitoes, stochastic drift) could influence *Wolbachia* loss. Again, in the paper Pavan et al. 2025 (ref #42), we tested the impact of external variables - air temperature, change to spinosad (lagged by 4 months), land temperature (LST), NDVI, presence of other mosquitoes, rainfall - as inferred from the MCMC simulations, see below.

**Fig. 9:** 

Full data is available at figshare: https://doi.org/10.6084/m9.figshare.27477987.v1 allowing further analysis. But looking for all the evidence available and data analysis, we hypothesized the increase in Spinosad coverage reduced mosquito population, both *Wolbachia*-infected and -uninfected, in the study area, but only the latter could recover when larvicide application ceased due to a well-known fitness cost in egg hatching due to *Wolbachia* presence.

5) Interpretation of epidemiological impact

• The link to Rio's 2024 dengue epidemic is tenuous, as Wolbachia releases covered 7 of the city. The text should avoid implying causation at this scale. The current framing is overly confident and risks overstating public health impact.

**A:** We appreciate Reviewer observation. We have edited different sections of the Discussion to avoid cause-effect relations between dengue epidemic in 2024 and the drop in Wolbachia frequency. The most critical section now reads: "An additional consequence of suboptimal *w*Mel frequencies in the field is the reduced level of community protection, as lower prevalence of *Wolbachia* is expected to result in diminished interruption of arbovirus transmission. Notably, in 2024, Rio de Janeiro experienced its most severe dengue outbreak in the past decade.^(66)^ Clearly, the reported increase in dengue transmission is not directly related to the drop in *Wolbachia* frequency given it covers less than 7% of the city's total area. However, spatiotemporal Bayesian models were fit to estimate the effect of introgression on dengue incidence during 2020-2024. ^(67)^ Authors observed the *Wolbachia* deployment was not associated with reductions in dengue incidence in 2024, amid a large dengue epidemic was reported in Rio de Janeiro city. ^(67)^ Moreover, introgression >45% was associated with increased dengue incidence. ^(67)^ The updated frequency of *w*Mel in Rio de Janeiro revealed all 12 neighborhoods sampled had introgression levels below the 45% threshold suggested. ^(67)^"

6) Figures and clarity

• Figure 5: Barplots of Wolbachia prevalence lack confidence intervals. Given small sample sizes, CIs (e.g., binomial or Wilson) are essential to show uncertainty. Without them, differences may appear more robust than they are.

**A:** We agree with Reviewer recomendation and we have produced new graphs, more informative. As we stated above, we selected three timepoints covering a total of three years of monitoring:

November 2021 to represent the beginning of the mosquito season in the city and when wMel frequency peaked.

February/2023, to represent an intermediate point of the timeseries considering its 36-month range.

Nov/2024 The last time point represents the most recent sampling, exactly 30 months after the population crash.

We reformulated the figures to better represent the spatiotemporal fluctuation in the wMel frequency observed. We adopted error bars in the new Figure 4A.

• I assume that in Figure 6: "NO Wolbachia" means individuals without Wolbachia infection. Yet, this group is shown with positive values for the loads. This is confusing. The authors must clarify how infection status was defined, why density values are shown for negatives, and whether these represent assay noise, sub-threshold infections, or plotting artefacts.

**A:** We have edited the figure.

• qPCR output plots currently in the main figures could be moved to Supplementary Data, with only summary results retained. This would streamline the main text.

**A:** We agree with reviewer recommendation and moved the qPCR plots to supplementary data.

7) Programmatic implications

• The conclusion that Wolbachia must be "reinforced before expanding elsewhere" is sensible, but currently too general. What prevalence threshold is considered protective? Explicitly linking findings to WHO guidelines or programmatic targets would increase the utility of the recommendations.

**A:** We agree with Reviewer in regards of the prevalence threshold that is considered protective. However, there is currently strong debate to define it. The work presented under reference #67 fitted spatiotemporal Bayesian models to estimate the effect of introgression on dengue incidence during 2020-2024. Authors observed strong association between dengue incidence and wMel introgression below 45%. We have added the following sentences to the Discussion section: "The prevalence threshold of *w*Mel to effectively protect local communities is still unknown, but it must consider the *Wolbachia* strain, the spatiotemporal heterogeneity of its frequency, herd immunity, and dengue serotype, to mention a few. A preliminary result showed that introgression <45% was associated with increased dengue incidence during 2024 in Rio de Janeiro city. ^(67)^ However, further investigations are required to understand whether this threshold can be extrapolated elsewhere."

Minor Comments

1) Abstract: rephrase "Regardless the wMel infection status" → "Regardless of the wMel infection status."

2) Introduction: More clearly connect the cited heterogeneity studies (refs 16-20) to Rio's unique context.

3) Methods: Provide more detail on neighborhood selection and improve Figure 1 map.

4) Results: Present neighborhoods with zero wMel in tabular form for clarity.

5) Discussion: Expand the point on seasonal thermal stress and its likely contribution to Wolbachia instability in Rio.

**A:** Done as recommended.

Recommendation: Major revision.

The study is potentially valuable but is currently weakened by limited spatial presentation, lack of molecular assay validation, and overconfident causal claims. Addressing these issues with additional data, more cautious interpretation, and clearer figures will be necessary for publication.

**A:** We appreciate the Reviewer comments on the first version of this manuscript. We hope the edited version has solved the concerns raised.

Reviewer: 2

This paper examining Wolbachia establishment in Rio de Janeiro for dengue control is a little confusing in its framing and the conclusions it draws from very limited data. The general narrative is that Wolbachia has somehow been established and then lost from mosquito populations in Rio and this is somehow connected to the ecological perturbation of using Spinosad insecticides. Everything is presented as correlation without demonstration of causality. I think a much simpler explanation for the observed data is that Wolbachia establishment never succeeded in Rio initially. Successful introgression requires the Wolbachia frequency to exceed the unstable point as defined by the models of Michael Turelli. The reported Wolbachia frequencies in Rio -- around 30-40% are very close to the measured unstable point of approx. 0.3 as measured in Cairns, Australia for the wMel strain. Given this closeness I think a more likely and simpler explanation than invasion followed by Wolbachia loss is that invasion was never accomplished (as defined by robustly exceeding the unstable point). Turelli's models predict that the closer the Wolbachia frequency is to the unstable point the slower the introgression dynamics will be -- either for invasion or loss and indeed what appears to be happening in Rio is a gradual loss of infection, consistent with the modelling, and this loss is happening slowly as predicted. The issue of the Spinosad insecticide application having some causal link with this loss seems highly unlikely as both populations of Wolbachia infected and uninfected mosquitoes are expected to be equally susceptible to the insecticide and so it should not be important in influencing the introgression dynamics. So, in my opinion the authors are trying to weave a complex narrative around something that is much simpler, and that is that Wolbachia was never successfully established and is slowly decaying in frequency in these areas. There is no need to invoke more complicated explanations and in fact this could be quite misleading. The key point as mentioned by authors is that in adjoining neighbourhoods in Niteroi, the introgression is complete and stable and experiences the same weather conditions as Rio, arguing against a general climate issue affecting introgression. Interestingly, when releases were first undertaken in Jurajuba, in Niteroi, there was initial difficulty in establishing Wolbachia, requiring re-releases but once established it was extremely robust.

So, while the paper attempts to make the claim that complex environments might lead to loss of Wolbachia a better conclusion in my opinion is that complex environments may make establishment of Wolbachia more challenging. Indeed, in this setting there were poorer neighbourhoods that routinely experience elevated levels of violence, interrupting release activities for weeks at a time during deployment. These disruptions together with possible underdosing may be much more likely to explain the introgression dynamics. While initial dose can be an important consideration, we also need to consider the role of the unhatched egg bank in certain settings which will influence establishment dynamics and could be an important but relatively unexplored critical element of the mosquito ecology that will influence Wolbachia establishment. Indeed, it may have been a critical element explaining the dynamics of Wolbachia in Jurajuba, Niteroi. Considering the novelty of the intervention I think it is normal that as we undertake deployments at increasing scale in a Public Health context we can expect some failures and optimisation that will be required in learning the best ways to deploy. However, that is very different from thinking that the method is inherently unstable. The success in Niteroi is evidence against the latter conclusion.

**A:** We appreciate Reviewer observations. Although we believe most of the key data requested is available in the publication in which we demonstrated the collapse of *Wolbachia* after individually screening by RT-qPCR wMel presence in 7,613 field-collected *Ae. aegypti* (Pavan et al. 2025). The aim of the manuscript under evaluation is to share an update of *w*Mel frequency exactly 30 months after it crashed due to an increase in coverage of the larvicide Spinosad in the study area.

We acknowledge the fact this manuscript is an update of the actual *Wolbachia* frequency was not properly described. Therefore, we edited several sections of the manuscript to make it clearer in the second version, and created a new Figure with a timeline of Rio de Janeiro large-scale deployments, from 2019-2025.

Regarding the very important point raised about whether *Wolbachia* establishment suceeded or not, previous work by Ribeiro-dos-Santos et al. (2022) showed a dengue reduction of 38% and 10% in dengue and chikungunya transmission in the areas with *Wolbachia*. Although it is definitely a lower protective effect than achieved to Niterói, it seems to strong and somehow not fair to mention its introgression has never suceeded. After all, if there is no succesful invasion there should be no protective effect from it. Due to massive literature available from Brazil and other endemic sites, we strongly believe *Wolbachia* worked in Rio but at suboptimal levels. And part of our ongoing research is to understand the reasons why it worked poorly in Rio. The concept that "invasion was never accomplished" pointed ou by reviewer, is controversial. *Wolbachia* deployment ended by the World Mosquito Program at December 2019 and the wMel strain frequency remained reasonably stable until May 2023. If *Wolbachia* invasion was never accomplished, as stated by Reviewer, how could we explain its frequency maintenance for 3.5 years without additional releases?

Turelli's model has been largely invocated to define *Wolbachia* dynamics into its hosts. However, as any other model, it does not fully represents reality. For instance, the theory underlying the bistable equilibrium states *Wolbachia* should invade or fail. However, we have very compeiling evidence showing the co-existence of *Wolbachia*-infected and -uninfected individuals, both at a smaller spatial scale but also at a larger area, references #16 and #42, respectively. As mentioned before, the increase in Spinosad coversage during the summer of 2022 overlaps with the mosquito season, but this year we had very few mosquito population in the study areas, meaning both *Wolbachia*-infected and -uninfected were affected. The negative effect of *Wolbachia* on egg-hatching is well-documented and it implies the *Wolbachia*-infected mosquitoes do not have an egg bank to recover after a significative suppression in adult population takes place. But this is a story already published in Pavan et al. (2025). The manuscript herein deals only with an update frequency of wMel in the same area of Rio de Janeiro, providing thus a current snapshot about *Wolbachia* frequency.

# Reviewer report: REVIEWERS' COMMENTS

## REVIEWER #1

I am not satisfied with the response of the authors on my prior comments. My expectation is that this alternative hypothesis which can also explain the observations be included in the discussion as an alternative hypothesis for the data. Wolbachia establishment (meant as introgression that is stable) has not been met and the possible positive epidemiological impact is expected and not relevant to the question of whether the Wolbachia infection is stable or not. I don't think this alternative view can be so easily ignored to support the over dramatised conclusion drawn from the current version.

In relation to the epidemiology it is also notable that a key paper on the proposed impact of Wolbachia on disease impact is missing and I believe would strengthen the paper if it was cited and discussed. Ferguson et al Science Translational Medicine 2015 (DOI: 10.1126/scitranslmed.3010370) shows that the epidemiological impact of Wolbachia is not absolute but represents a reduction in the effective R0 which in situations where the R0 is moderate will lead to elimination, but when the R0 is extremely high as was seen in Brazil in 2024 transmission reduction will not lead to disease elimination, but still produce a drop in disease. The paper would be strengthened by including this nuance in the discussion in relation to the epidemiology.

I also believe the overly dramatic word "crash" in the title is misleading and instead should be replaced with "introduction".

## AUTHORS' RESPONSE TO THE REVIEWERS

Reviewer: 1

I am not satisfied with the response of the authors on my prior comments. My expectation is that this alternative hypothesis which can also explain the observations be included in the discussion as an alternative hypothesis for the data. Wolbachia establishment (meant as introgression that is stable) has not been met and the possible positive epidemiological impact is expected and not relevant to the question of whether the Wolbachia infection is stable or not. I don't think this alternative view can be so easily ignored to support the over dramatised conclusion drawn from the current version.

**A:** We thank the reviewer for raising this important point and for encouraging consideration of alternative interpretations. However, the temporal dynamics observed in Rio de Janeiro points a much more complex scenario than suggested by mathematical modeling and data from elsewhere (Australia and Southeast Asia). The bistable equilibrium suggested by *Wolbachia* modeling papers (Hoffmann et al. 1990; Barton & Turelli 2011) is challenged by field data that reveals introgression is not a binomial outcome, but an index that fluctuates over space and time, as revealed by a careful observation in public available data from Rio de Janeiro and Niteroi (Brazil: Garcia et al. 2019; Pinto et al. 2021; Ribeiro-dos-Santos et al. 2022). This scenario has been commonly described as "incomplete introgression", as pointed out by authors associated with the World Mosquito Program (Ribeiro-dos-Santos et al. 2022), presenting moderate effect on dengue incidence. Remarkably, full introgression (commonly pointed as >60%) has been observed and associated with strong impact on dengue incidence in Yogyakarta (Indonesia: Utarini et al. 2021), Bello, Medellin and Itaguí (Colômbia: Velez et al. 2023), and Townsville (Australia: O'Neill et al. 2019).

Specifically on Rio de Janeiro. After the completion of wMel releases in December 2019, wMel frequencies remained relatively stable (30-50%) from January 2020 to April 2022, a period comprised of 28 months. From our perspective, a clear case of "incomplete introgression" as classified by Ribeiro-dos-Santos et al. (2022). Nevertheless, the infection frequency persisted at stable levels for 28 months in the field after the cessation of releases.

Previous mark-release-recapture studies in Rio de Janeiro demonstrated an average life expectancy of female *Aedes aegypti* of ~15 days (Maciel-de-Freitas et al. 2007). Therefore, this period roughly corresponds to around 56 mosquito generations without additional wMel releases. Persistence across such many generations strongly suggests that Wolbachia introgression had reached a stable although incomplete introgression prior to a disturbance that was observed in April 2022.

Regarding the epidemiological outcome of Wolbachia deployment. Notably, previous analyses conducted in Rio de Janeiro (Santos et al. 2022; DOI: 10.1016/S1473-3099(22)00436-4) reported a similar pattern of incomplete introgression, with wMel frequencies remaining around 30-40% between August 2017 and December 2019. Despite this intermediate level of introgression, Wolbachia deployments were associated with a 38% reduction in dengue incidence and a 10% reduction in chikungunya incidence. These findings suggest that even moderate wMel frequencies may contribute to reductions in arbovirus transmission at the population level. A comparable pattern was also observed in Zone 3 of Niterói during the 2017-2019 releases (Pinto et al., 2021; DOI: 10.1371/journal.pntd.0009556). These observations suggest that the scenario observed, characterized by incomplete yet temporally stable wMel introgression persisting for several years after the cessation of releases, may represent a realistic outcome in large and environmentally heterogeneous urban settings. Such dynamics may therefore occur in other Brazilian regions where this technology is currently being implemented or planned and should be considered when evaluating the long-term performance of Wolbachia-based interventions, rather than interpreting intermediate frequencies as a failure of establishment.

For comparison, a contrasting outcome consistent with failure of wMel establishment was reported by Garcia et al. (2019) - DOI: 10.1371/journal.pntd.0007023. Following the first round of wMel releases in Tubiacanga (Rio de Janeiro), wMel frequency peaked at approximately 65% at the end of the release period but declined rapidly to ~10% within 04 months after releases ceased. This rapid decline indicates a lack of persistence of the infection in the field once releases stopped. In contrast, in the present scenario in Rio de Janeiro, wMel frequencies remained relatively stable at approximately 30-40% for about 28 months after the end of releases, indicating a markedly different temporal dynamic.

Regarding discussing hypothesis for the crash observed in Wolbachia wMel strain In Rio, it was exhaustively debated in Pavan et al. (2024). It is also important to clarify that the objective of the present paper is not to investigate the mechanisms responsible for the rapid decline in wMel frequency observed in Rio de Janeiro in April 2022. The description of the longitudinal sampling and the hypothesis in which the *w*Mel crash was due to the high coverage of the biolarvicide Spinosad was explored in the paper published by Pavan et al. (2024). In this manuscript, our aim is solely to provide an update on *Wolbachia* frequency after the end of the monitoring reported in Pavan et al. (2024).

Hoffmann, A. A., Turelli M., and Harshman L. G. 1990. Factors affecting the distribution of cytoplasmic incompatibility in *Drosophila simulans*. Genetics 126:933-948.

Barton, N. H., and Turelli M. 2011. Spatial waves of advance with bistable dynamics: cytoplasmic and genetic analogues of Allee effects. Am. Nat. 178:E48-E75.

Garcia G de A, Sylvestre G, Aguiar R, da Costa GB, Martins AJ, Lima JBP, et al. Matching the genetics of released and local *Aedes aegypti* populations is critical to assure *Wolbachia* invasion. PLoS Negl Trop Dis. 2019;13: e0007023.

Pinto SB, Riback TIS, Sylvestre G, Costa G, Peixoto J, Dias FBS, et al. Effectiveness of *Wolbachia*-infected mosquito deployments in reducing the incidence of dengue and other *Aedes*-borne diseases in Niterói, Brazil: A quasi-experimental study. PLoS Negl Trop Dis. 2021;15: e0009556.

Ribeiro dos Santos G, Durovni B, Saraceni V, Souza Riback TI, Pinto SB, Anders KL, et al. Estimating the effect of the *w*Mel release programme on the incidence of dengue and chikungunya in Rio de Janeiro, Brazil: a spatiotemporal modelling study. Lancet Infect Dis. 2022;22: 1587-1595.

Utarini A, Indriani C, Ahmad RA, Tantowijoyo W, Arguni E, Ansari MR, et al. Efficacy of *Wolbachia*-Infected Mosquito Deployments for the Control of Dengue. New England Journal of Medicine. 2021;384: 2177-2186.

Velez ID, Uribe A, Barajas J, Uribe S, Ángel S, Suaza-Vasco JD, et al. Large-scale releases and establishment of *w*Mel *Wolbachia* in *Aedes aegypti* mosquitoes throughout the Cities of Bello, Medellín and Itagüí, Colombia. PLoS Negl Trop Dis. 2023;17: e0011642.

O'Neill SL, Ryan PA, Turley AP et al. Scaled deployment of Wolbachia to protect the community from dengue and other Aedes transmitted arboviruses. Gates Open Research 2019, 2:36

Maciel-de-Freitas R, Codeço CT, Lourenço-de-Oliveira R. Daily survival rates and dispersal of Aedes aegypti females in Rio de Janeiro, Brazil. Am J Trop Med Hyg 2007 Apr;76(4):659-65.

Pavan MG, Gnonhoue FJ, Corrêa-Antônio J, Padilha KP, Garcia GA, Oliveira F, Brito LP, Martins AJ, Corbel V, Lima JBP, Wallau GL, Hoffmann AA, Cruz OG, Villela DAM, Maciel-de-Freitas R. The long-term persistence of the *w*Mel strain in Rio de Janeiro is threatened by poor integrated vector management and bacterium fitness cost on *Aedes aegypti*. PLoS Negl Trop Dis 19(7): e0013372

In relation to the epidemiology it is also notable that a key paper on the proposed impact of Wolbachia on disease impact is missing and I believe would strengthen the paper if it was cited and discussed. Ferguson et al Science Translational Medicine 2015 (DOI: 10.1126/scitranslmed.3010370) shows that the epidemiological impact of Wolbachia is not absolute but represents a reduction in the effective R0 which in situations where the R0 is moderate will lead to elimination, but when the R0 is extremely high as was seen in Brazil in 2024 transmission reduction will not lead to disease elimination, but still produce a drop in disease. The paper would be strengthened by including this nuance in the discussion in relation to the epidemiology.

**A:** We thank the reviewer for this valuable suggestion. We agree that this study provides important context for interpreting the expected epidemiological impact of *Wolbachia* deployments. Following the reviewer's recommendation, we have incorporated the reference Ferguson et al. (2015) into the Discussion and expanded the text to emphasize that *Wolbachia* is expected to reduce the effective reproductive number (R₀), rather than completely block transmission. This point has now been incorporated into the Discussion before addressing the epidemiological patterns observed in Rio de Janeiro.

I also believe the overly dramatic word "crash" in the title is misleading and instead should be replaced with "introduction".

**A:** We thank the reviewer for this comment. We have now replaced the word "crash" with "decline" in the title of the manuscript. However, the term "crash" was originally introduced in Pavan et al. (2024), where the authors described and discussed the rapid decline in wMel frequency observed in Rio de Janeiro and explored the potential mechanisms underlying this pattern. Because the present study builds directly upon the scenario described in that previously published work, we opted to maintain the same terminology to ensure consistency with the existing literature.

In addition, the study done by Pavan et al (2024) has already been cited by subsequent publications that refer to the same system and context (e.g., Corrêa-Antônio et al. 2025, DOI: 10.1038/s42003-025-08862-7; Corbel et al. 2025, DOI: 10.1371/journal.pntd.0013675; Anders et al. 2025, DOI: 10.3390/tropicalmed10090237; de Morais Batista et al. 2025, DOI: 10.1016/j.lana.2025.101327). Therefore, rather than sound "over dramatized", we believe that maintaining the terminology helps preserve continuity with the emerging literature discussing Wolbachia dynamics in Brazilian settings, besides preserving the seriousness required in investigations in the field of public health.

# Reviewer report: REVIEWERS' COMMENTS

## REVIEWER #1

No comments.

## DECISION

DECISION: ACCEPT SUBMISSION | RECEIVED 15 AUGUST 2025 | ACCEPTED 05 MAY 2026
